# Computational model predicts the neural mechanisms of prepulse inhibition in *Drosophila* larvae

**DOI:** 10.1038/s41598-022-19210-8

**Published:** 2022-09-08

**Authors:** Kotaro Furuya, Yuki Katsumata, Masayuki Ishibashi, Yutaro Matsumoto, Takako Morimoto, Toru Aonishi

**Affiliations:** 1 School of Computing, Tokyo Institute of Technology, 4259 Nagatsuta-cho, Yokohama-shi, Kanagawa 226–8503 Japan; 2 School of Life Sciences, Tokyo University of Pharmacy and Life Sciences, 1432–1 Horinouchi, Hachioji-shi, Tokyo 192–0392 Japan

**Keywords:** Diseases of the nervous system, Autism spectrum disorders, Sensorimotor processing, Neuroscience, Computational neuroscience, Psychiatric disorders

## Abstract

Prepulse inhibition (PPI) is a behavioural phenomenon in which a preceding weaker stimulus suppresses the startle response to a subsequent stimulus. The effect of PPI has been found to be reduced in psychiatric patients and is a promising neurophysiological indicator of psychiatric disorders. Because the neural circuit of the startle response has been identified at the cellular level, investigating the mechanism underlying PPI in *Drosophila melanogaster* larvae through experiment-based mathematical modelling can provide valuable insights. We recently identified PPI in *Drosophila* larvae and found that PPI was reduced in larvae mutated with the *Centaurin gamma 1A* (CenG1A) gene, which may be associated with autism. In this study, we used numerical simulations to investigate the neural mechanisms underlying PPI in *Drosophila* larvae. We adjusted the parameters of a previously developed *Drosophila* larvae computational model and demonstrated that the model could reproduce several behaviours, including PPI. An analysis of the temporal changes in neuronal activity when PPI occurs using our neural circuit model suggested that the activity of specific neurons triggered by prepulses has a considerable effect on PPI. Furthermore, we validated our speculations on PPI reduction in CenG1A mutants with simulations.

## Introduction

With the number of patients increasing yearly, psychiatric disorders are an important type of brain disorder that must be investigated. However, the wide range of symptoms of psychiatric disorders increases the difficulty of diagnosis. To elucidate the molecular mechanisms of these diseases and develop treatment methods, animal experiments are essential. Therefore, behavioural indicators that are common to several animals, including humans, are useful. Prepulse inhibition (PPI) is a behavioural phenomenon that has attracted increasing attention as such an indicator. In this phenomenon, the startle response caused by a startle stimulus such as an air puff or a loud sound is suppressed when it is directly preceded by a weaker stimulus (prepulse)^1^. PPI is reduced in patients with psychiatric disorders such as schizophrenia^1–4^ and is considered to be an effective endophenotypic candidate for schizophrenia^5,6^. This is further supported by showing the positive/negative symptoms of schizophrenia are correlated with reduction of PPI baseline^7,8^ and attentional modulations of PPI^9–12^. Various psychiatric disorders have been associated with PPI, including autism spectrum disorder^13,14^, Asperger syndrome^15^, bipolar disorder^16^, Tourette syndrome^17^, obsessive–compulsive disorder^18^, and posttraumatic stress disorder^19^. Although the relationship between PPI and various psychiatric disorders has been thoroughly reported and studied, the mechanisms underlying PPI and the deficiency of PPI in psychiatric disorders remain unclear. Therefore, investigations into the neural mechanisms associated with PPI and the molecular mechanisms underlying the reduction in PPI in patients with psychiatric disorders are critical for understanding these disorders.

PPI is considered to be a measure of sensorimotor gating. Sensorimotor gating is defined as the state-dependent regulation of the transmission of sensory information to a motor system, which allows relevant information to be processed selectively and efficiently^6^. Although PPI has been observed for various types of sensory stimulations, including vision, hearing, and touch^20,21^, auditory stimuli are most often used in experiments. Recently, the neural circuits underlying PPI have been identified in rats^22^. In humans, positron emission tomography (PET) and anatomical/functional magnetic resonance imaging (MRI) have been used in PPI research, showing that frontal-striatal-thalamic circuit is involved in PPI^6^. One of the most important factors in modulating PPI is the lead interval. The lead interval is the time interval between a prepulse and the subsequent pulse. The effect of PPI depends on the lead interval, and PPI has been reported to be reduced when the lead interval is too short or too long^23–25^. Thus, plots of the percentage of the startle response with PPI as a function of the lead interval are typically inverted U-shapes. Recently, based on PPI experimental data and the neural circuits associated with PPI, mathematical studies have attempted to develop models that reproduce PPI and its features and predict the underlying neural circuit mechanism^26–28^. However, there are considerably fewer mathematical studies on PPI than neurophysiological studies, and thus, the mechanism of PPI at the cellular level remains largely unknown. PPI occurs not only in primates but also in mammals, such as mice, and invertebrates, such as the model organism *Tritonia diomedea*^29,30^. Recently, we reported for the first time PPI in the larvae of the model organism *Drosophila melanogaster*^31^. In *Drosophila*, the neural circuits involved with various behaviours can be identified at the cellular level, allowing circuit models to be developed based on a connectome and mathematical explorations to be performed. In this paper, we conducted mathematical research on PPI in *Drosophila* larvae.

**Figure 1:**
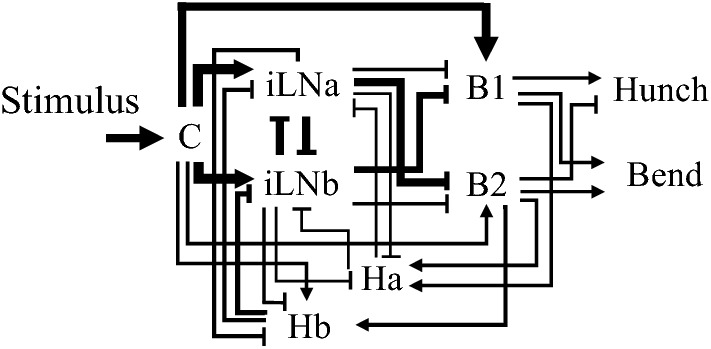
Schematic of the reconstructed Basin circuit. The edge width increases with the number of synapses. The sharp arrowheads indicate excitatory connections, while the square arrowheads indicate inhibitory connections. C: mechano-sensory chordotonal neurons, B1: Basin-1 neuron, B2: Basin-2 neuron, iLNa: inhibitory local interneurons-a, iLNb: inhibitory local interneurons-b, Ha: Handle neuron-a, Ha: Handle neuron-b. Reproduced from Jovanic et al*.*^32^ Fig. 2e with permission from Elsevier.

Our previous study of PPI used the acoustic startle response paradigm in *Drosophila* larvae. The neural circuit associated with this startle response was identified by Jovanic et al*.*^32^. They used electrophysiological recordings and genetic manipulations to study the neural mechanisms of the startle response to air puffs in *Drosophila* larvae. *Drosophila* larvae exhibit two startle behaviours: a “hunch”, in which a larva retracts its head, and a “bend”, in which a larva bends its body. Jovanic et al*.*^32^ studied the mechanism underlying neural circuits that evoked either hunching or bending behaviour in response to the same air puff stimulus. They identified neural networks related to behaviour selection and constructed a neural circuit model based on the connectome through electron microscopy (Fig. 1). They used this neural circuit model to simulate temporal changes in the activity level of neuronal cell groups associated with the startle response and reproduced experimental results of startle responses.

Here, we sought to qualitatively reproduce the PPI of the acoustic startle response paradigm in *Drosophila* larvae by using the above neural circuit model. Furthermore, we used a computational model to determine the reason for the reduction in PPI in *Centaurin gamma 1A* (CenG1A) mutants. CenG1A belongs to the Centaurin family of proteins, which has been linked to autism^33^. CenG1A contains several functional domains, including an ADP ribosylation factor (Arf, a small G-protein) GTPase-activating protein (GAP) domain. We reported that CenG1A can act as a negative regulator of neurotransmitter release^34^. We found that the PPI response is decreased in larvae with suppressed CenG1A function (CenG1A mutants)^31^. In Jovanic et al*.*^32^ neural circuit model, except for the synaptic connectivity reconstructed by electron microscopy, all parameters for reproducing the startle response were set heuristically. First, we sought to reproduce PPI in numerical simulations by adjusting these heuristically determined parameters while using other parameters that were determined based on Jovanic et al*.*^32^ experimental findings. In this process, we identified the neural circuit features necessary for inducing PPI and proposed a mathematical model for the neural mechanism underlying PPI. Second, we proposed a location in the neural circuit related to PPI reduction in CenG1A mutants. Thus, we applied a cellular-level neural circuit model to elucidate neural circuit features that allow experimental results to explain the neural mechanism of PPI. To the best of our knowledge, this is the first study to replicate multiple behavioural experiments and explain the reduction in PPI associated with psychiatric disorders in the same model.

## Methods

### Behavioural experiment

**Figure 2:**
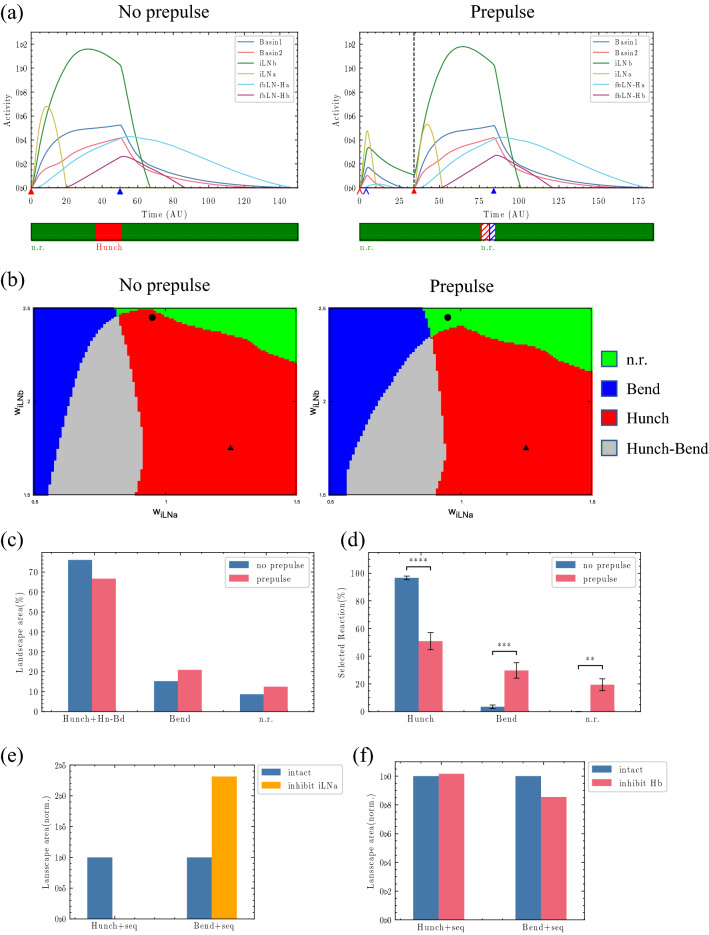
Reproduction of the behavioural experiment using the neural circuit model with adjusted parameters. (**a**) Temporal changes in the activity of each neuron at the black dot (${\mathrm{w}}_{\text{iLNa}}=0.95,{\mathrm{w}}_{\text{iLNb}}=2.45$), where the suppression of the startle response occurs due to PPI in (**b**). Left: Without a prepulse; Right: With a prepulse. The vertical dashed black line indicates the time of the pulse input. The thin arrow head pointing to the horizontal axis indicates the prepulse timing, and the thick arrow head indicates the pulse timing. The red and blue colours indicate the onset and offset times, respectively. The colours in the band below each graph indicate the behaviour at the corresponding time. Green: n.r., red: hunch, blue: bend. The behaviours shown with diagonal lines are classified as n.r. because their durations are shorter than the cut-off time (10 time units). (**b**) Behavioural landscape in the ${\mathrm{w}}_{\text{iLNb}}- {\mathrm{w}}_{\text{iLNa}}$ space. Left: Without a prepulse. Right: With a prepulse. (**c**) Changes in the percentage of each behaviour in the behavioural landscape due to prepulses. The Hunch + Hn-Bd indicates the sum of the hunch and the hunch-bend sequence because we counted the first selected behaviour in our experimental procedure. The bend-hunch sequence did not occur. (**d**) Experimental results of behavioural change due to prepulses. (**e**) iLNa inhibition results obtained with the model used in this study. The Hunch (Bend) + seq includes the Hunch (Bend) and both sequences, as in Jovanic et al*.*^32^ (**f**) Hb inhibition results obtained with the model used in this study.

The methods used in the behavioural experiment shown in Fig. 2d are described in Matsumoto et al*.*^31^. However, in Matsumoto et al*.*^31^, hunching and bending were not distinguished but instead considered together as the startle response. We conducted behavioural experiments using wild-type *Drosophila melanogaster Canton-S* and analysed the types of behaviours (Fig. 2d). All experimental conditions, including the auditory stimulations, were the same as those previously described^31^. Briefly, the pulse amplitude was 75 dB, the pulse duration was 500 ms (for the pulse) or 40 ms (for the prepulse), and the interpulse interval was 300 ms. The sounds were generated with natural recordings of wasps taken from the Jungle Walk website, as described in Zhang et al*.*^35^, and modified using WavePad software (NCH Software, Greenwood Village, USA). The sound stimuli were processed in the neural network via the chordotonal organ, similar to the air puff stimuli in Jovanic’s study^31,32,35^. Hunch and bend are characteristic behaviours and can easily be discriminated by observing the behaviour of the larvae. We recorded videos and scored the behaviours off-line. We investigated the observed behaviour just after the sound stimulus was presented (within less than 1 s). When a sound stimulus was applied, a larva’s response of contracting its body was considered a “hunch,” while a response of bending its body left or right was considered a “bend.” If the larva did not respond, the behaviour was classified as “no reaction” (n.r.). Thus, behaviours were categorized into three types of responses. Ten larvae were placed on each agar plate. The stimulation was applied 10 times, and the percentage of each larval behaviour (hunch, bend, or no reaction) was determined by the 10 responses to these 10 stimulations. Experiments were performed on three or four dishes, and the data of 30–40 larvae were averaged. We denote this averaged percentage as the “selected reaction (%)” in Fig. 2d. Larvae that did not move at all during the experiment were not counted. We described how we calculated the startle response in our previous study in detail^31^. Briefly, the larval response to the pulse was scored as two points (strong startle response), one point (slight startle response), or zero points (no startle response). We then calculated the total points for one sound (one trial) for 10 larvae and the ratio against the full score (2 points × 10 larvae = 20 points). This ratio was defined as the startle response value for one trial. To determine the startle response value for each condition, the startle response value was averaged across 15–25 trials using 30–50 larvae on 3–5 plates. The larvae were raised at 22 °C, which is the same temperature as used in previous studies. The larvae were collected 4–5 days after egg laying, at which point they were living in the food and had not yet reached the wandering stage, for use in the experiments. These experiments were approved by the institutional and licensing committee with protocol number LSR3-012 and were performed in accordance with relevant named guidelines and regulations.

For additional experimental data to compare with the simulation results, with the exception of the data shown in Fig. 2d, we used behavioural experimental data taken from Matsumoto et al*.*^31^ and Jovanic et al*.*^32^.

### Mathematical model

We used the simple rate model proposed by Jovanic et al*.*^32^. The model describes the neural pathway involved in larva’s startle response evoked through chordotonal organs. This neural circuit consists of mechano-sensory chordotonal neurons (Ch), feedforward inhibitory local interneurons (iLNa and iLNb), basin projection neurons (Basin-1 and Basin-2) and handle neurons (Ha and Hb) (Fig. 1). Stimuli are received by Ch which transmits excitatory inputs to iLNa and iLNb and strong and weak excitatory inputs to Basin-1 and Basin-2, respectively. iLNa and iLNb form inhibitory connections to each other, and they form feedforward inhibitory connections to Basin-1 and Basin-2. Especially, iLNa more strongly inhibits Basin-2 than Basin-1, while iLNb more strongly inhibits Basin-1 than Basin-2. From this circuit specification, iLNa and iLNb act competitively through reciprocal inhibition to each other, and the activity of iLNa and iLNb has a significant effect on Basin-1 and Basin-2 in determining behaviour. If iLNa is strongly active, iLNb and Basin-2 are strongly inhibited. Thus, iLNa exceeding iLNb causes the inhibition of Basin-2 and the disinhibition of Basin-1. Oppositely, iLNb exceeding iLNa causes the inhibition of Basin-1 and the disinhibition of Basin-2. Moreover, Basin-1 and Basin-2 form a feedback pathway to themselves via Ha and Hb. Ha and Hb, which have excitatory connections from Basin-1 and Basin-2, form reciprocal inhibitory connections with iLNa and iLNb. Thus, the activity of Basin-1 and Basin-2 are regulated through the balance between the activities of feedforward inhibitory local interneurons and handle neurons. A more thorough discussion of this circuit can be found in Jovanic et al*.*^32^.

Reducing each neuron category to a single node, the activity of each neuron in the circuit is given by the following formula:

$${\uptau }_{i} \frac{{dr_{i} }}{dt} = - {\text{V}}_{0,i} - r_{i} + s_{i} + \left( {r^{{{\text{max}}}} - r_{i} } \right)\mathop \sum \limits_{j = 1}^{7} {\text{A}}_{ij}^{{{\text{ex}}}} r_{j} - \mathop \sum \limits_{j = 1}^{7} {\text{A}}_{ij}^{{{\text{in}}}} r_{j} \,\left( {i = 1, \ldots ,7} \right)$$

where ${\uptau }_{i}$ is the time constant, ${r}_{i}\left(\ge 0\right)$ is the cell activity level, ${\mathrm{V}}_{0,i}$ is the threshold for activation, ${s}_{i}$ is the stimulus input, ${r}^{\text{max}}$ is the maximum value of $r$, and ${\mathrm{A}}_{\mathrm{ij}}^{\text{ex}}$ and ${\mathrm{A}}_{ij}^{\text{in}}$ are the excitatory and inhibitory connection strengths from neuron $j$ to neuron $i$, respectively. The subscripts here correspond to the neuron categories shown in Fig. 1 as follows: 1: Ch, 2: B1, 3: B2, 4: iLNb, 5: iLNa, 6: Ha, and 7: Hb. The units of time $t$ are arbitrary units (AU), as in Jovanic et al*.*^32^.

The connectivity matrices ${\mathrm{A}}^{\text{ex}}$ and ${\mathrm{A}}^{\text{in}}$ were set based on synaptic measurements conducted using electron microscopy. The matrices ${\mathrm{A}}^{\text{ex}}$ and ${\mathrm{A}}^{\text{in}}$ are given below:

$$\begin{aligned} & {\text{A}}^{{{\text{ex}}}} = \left[ {\begin{array}{*{20}l} 0 \hfill & {\quad 0} \hfill & {\quad 0} \hfill & {\quad 0} \hfill & {\quad 0} \hfill & {\quad 0} \hfill & {\quad 0} \hfill \\ 2 \hfill & {\quad 0} \hfill & {\quad 0} \hfill & {\quad 0} \hfill & {\quad 0} \hfill & {\quad 0} \hfill & {\quad 0} \hfill \\ 1 \hfill & {\quad 0} \hfill & {\quad 0} \hfill & {\quad 0} \hfill & {\quad 0} \hfill & {\quad 0} \hfill & {\quad 0} \hfill \\ {{\text{w}}_{{{\text{iLNb}}}} } \hfill & {\quad 0} \hfill & {\quad 0} \hfill & {\quad 0} \hfill & {\quad 0} \hfill & {\quad 0} \hfill & {\quad 0} \hfill \\ {{\text{w}}_{{{\text{iLNa}}}} } \hfill & {\quad 0} \hfill & {\quad 0} \hfill & {\quad 0} \hfill & {\quad 0} \hfill & {\quad 0} \hfill & {\quad 0} \hfill \\ 0 \hfill & {\quad 0.2} \hfill & {\quad 0.2} \hfill & {\quad 0} \hfill & {\quad 0} \hfill & {\quad 0} \hfill & {\quad 0} \hfill \\ {0.4} \hfill & {\quad 0} \hfill & {\quad 0.5} \hfill & {\quad 0} \hfill & {\quad 0} \hfill & {\quad 0} \hfill & {\quad 0} \hfill \\ \end{array} } \right], \\ & {\text{A}}^{{{\text{in}}}} = \left[ {\begin{array}{*{20}l} 0 \hfill & {\quad 0} \hfill & {\quad 0} \hfill & {\quad 0} \hfill & {\quad 0} \hfill & {\quad 0} \hfill & {\quad 0} \hfill \\ 0 \hfill & {\quad 0} \hfill & {\quad 0} \hfill & {\quad 1.06} \hfill & {\quad 0.83} \hfill & {\quad 0} \hfill & {\quad 0} \hfill \\ 0 \hfill & {\quad 0} \hfill & {\quad 0} \hfill & {\quad 0.6} \hfill & {\quad 3.55} \hfill & {\quad 0} \hfill & {\quad 0} \hfill \\ 0 \hfill & {\quad 0} \hfill & {\quad 0} \hfill & {\quad 0} \hfill & {\quad 2.02} \hfill & {\quad 1} \hfill & {\quad 1.31} \hfill \\ 0 \hfill & {\quad 0} \hfill & {\quad 0} \hfill & {\quad 1.63} \hfill & {\quad 0} \hfill & {\quad 0.66} \hfill & {\quad 1.98} \hfill \\ 0 \hfill & {\quad 0} \hfill & {\quad 0} \hfill & {\quad 1.1} \hfill & {\quad 0.67} \hfill & {\quad 0} \hfill & {\quad 0} \hfill \\ 0 \hfill & {\quad 0} \hfill & {\quad 0} \hfill & {\quad 1.04} \hfill & {\quad 7.86} \hfill & {\quad 0} \hfill & {\quad 0} \hfill \\ \end{array} } \right] \\ \end{aligned}$$

As in Jovanic et al*.*^32^ the magnitudes of w_iLNb_ and w_iLNa_, which are excitatory connections from C to iLNb and iLNa, have a range of values to represent differences in the activation of the two interneuronal classes. w_iLNb_ and w_iLNa_ ranged between 0.5–1.5 and 1.5–2.5, respectively. We assume that these differences result in the selection of distinct behaviours. Based on this assumption about individual differences, we can depict a behavioural landscape of behaviour selection in the w_iLNb_−w_iLNa_ space and evaluate the percentages of each behaviour. The dynamics were solved using the fourth-order Runge–Kutta method.

The experimental results of Jovanic et al*.*^32^ revealed that the behaviour selection of larvae is determined by Basin-1 and Basin-2 activity levels. Specifically, when Basin-1 is more active than Basin-2, the hunch behaviour is selected. When Basin-2 is more active than Basin-1, the bend behaviour is selected. When Basin-1 and Basin-2 are both weak, no reaction (n.r.) is selected. Accordingly, the conditional expressions for behaviour selection are given by

1 $$\begin{aligned} & {\text{Hunch}}:\frac{{\widehat{{r_{3} }}}}{{\widehat{{r_{2} }}}} < {0.8\,{\text{AND}}\,\widehat{{r_{2} }},\widehat{{r_{3} }}} > 0.5 \\ & {\text{Bend}}:\frac{{\widehat{{r_{3} }}}}{{\widehat{{r_{2} }}}} \ge 0.8\,{\text{AND}}\,\widehat{{r_{2} }},\widehat{{r_{3} }} > 0.5 \\ & {\text{n.r}}.:\widehat{{r_{2} }} \le 0.5\,{\text{AND}}\,\widehat{{r_{3} }} \le 0.5 \\ \end{aligned}$$

where $\widehat{{r}_{i}}={r}_{i}/{r}_{i}^{*}$. In this study, ${r}_{i}^{*}$ is the maximum value of ${r}_{i}$ for dynamics when ${s}_{1}=0.5, {\mathrm{w}}_{\text{iLNa}}=1, {\mathrm{w}}_{\text{iLNb}}=2.$ If hunching or bending changed to the other behaviour, we counted it as a sequence, that is, the hunch-bend sequence or the bend-hunch sequence.

As in Jovanic et al*.*^32^ behaviours that were extremely short in duration (the time during which the startle response’s conditional expression is satisfied) were not counted. In the analysis below, behaviours that were shorter in duration than a certain value were classified as n.r. A cut-off parameter was introduced as the behavioural duration threshold for classifying a behaviour as n.r. This value was set as 10 time units in the analysis below.

### Adjustment of parameters

The neural circuit model with Jovanic et al*.*^32^ parameters cannot reproduce PPI. They quantitatively configured the connectivity matrices ${\mathrm{A}}^{\text{ex}}$ and ${\mathrm{A}}^{\text{in}}$ with electron microscopy measurements and heuristically set the other parameters, which are shown in Table 1, to reproduce the startle response. Thus, in this study, we changed only these heuristic parameters and searched for new parameter values to reproduce both our PPI experimental results and Jovanic et al*.*^32^ experimental results. The parameter fitting was performed by changing the parameters individually and examining the effect of each parameter on the model. This method is more interpretable than other parameter search methods such as grid search. Ha and Hb have inhibitory connections to iLNa and iLNb. Therefore, their activation thresholds ${\mathrm{V}}_{\mathrm{0,6}}$ and ${\mathrm{V}}_{\mathrm{0,7}}$ were set to be greater than that of the other neurons, allowing prepulse effects to remain during subsequent stimulus inputs, resulting in PPI.

**Table 1:** Parameters.

	Jovanic et al*.*^32^	Our research
${\mathrm{V}}_{0}$	${\mathrm{V}}_{\mathrm{0,1}}=0,{\mathrm{V}}_{0,i}=10 (i > 1)$	${\mathrm{V}}_{\mathrm{0,1}}=0, {\mathrm{V}}_{0,i}=0.1 (2\le i\le 5),{\mathrm{V}}_{\mathrm{0,6}}=1,{\mathrm{V}}_{\mathrm{0,7}}=5$
${s}_{i}$	${s}_{1}=2.0,{s}_{i}=0 (i \ge 2)$	${s}_{1}\in \left[0,2.0\right],{s}_{i}=0 (i\ge 2)$
${r}^{\text{max}}$	$20$	$15$
${\uptau }_{i}$	${\uptau }_{i}=1,{\uptau }_{i}=35 (i\ge 2)$	${\uptau }_{i}=1,{\uptau }_{i}=30 (i\ge 2)$
Stimulus duration	$450$ time units	$50$ time units
Cut-off	$16$	$10$

Note that Jovanic et al*.*^32^ parameter values shown in Table 1 and the connectivity matrices shown in Sect. “Mathematical model” differ from the values given in their report. Because their values caused issues in our experiments, we confirmed the parameter values with the authors. The actual values they used in their numerical experiments are shown in this study.

## Results

### Reproduction of PPI

With the adjusted parameters, we conducted a numerical experiment using the same procedures as our PPI behavioural experiments. A prepulse was applied, followed by another pulse after a certain interval of time. The prepulse was shorter than the pulse to ensure it did not induce a startle response. The prepulse was applied for 4 time units. After an interval of 30 time units (i.e., the lead interval), the pulse stimulus was applied. In contrast to Jovanic et al*.*^32^ experiment, which used a direct air puff as the input stimulus, our experiment used sound from a speaker to evoke the startle response, which is a weaker stimulus than an air puff stimulus. As a result, in this simulation, the strength of the input stimulus to Ch neurons, ${s}_{1}$, is weaker than Jovanic et al.^32^ value and was set as 0.45. Figure 2a shows the typical behaviour of the circuit with the adjusted parameters under the condition of the startle response suppressed by a prepulse (${\mathrm{w}}_{\text{iLNa}}=0.95,{\mathrm{w}}_{\text{iLNb}}=2.45$). Figure 2a (left) shows the results without a prepulse, and Fig. 2a (right) shows the results with a prepulse. Without a prepulse, iLNa activity exceeded iLNb activity immediately after the stimulus was presented. Because iLNa inhibits Basin-2 and disinhibits Basin-1, Basin-1 was more active than Basin-2. This finding satisfied the condition shown in Eq. (1) and promoted a hunch response (left figure in Fig. 2a). In contrast, with a prepulse, because iLNb activity induced by the prepulse remained active until the pulse began, iLNb activity exceeded iLNa activity immediately after the pulse (the dotted line in the right figure of Fig. 2a) was presented. iLNb inhibited the activity of Basin-1, the difference in activity between Basin-1 and Basin-2 decreased, and the hunch response was suppressed, resulting in n.r. Although the peak of the difference between Basin-1 and Basin-2 did not change significantly, iLNb activity induced by the prepulse reduced the amount of time needed to satisfy the startle response condition, and the resulting duration was less than the cut-off parameter. Next, we simulated the ${\mathrm{w}}_{\text{iLNb}}- {\mathrm{w}}_{\text{iLNa}}$ space in 0.01 increments along both axes and determined which behaviours were selected for each combination. Figure 2b shows the behavioural landscape of the ${\mathrm{w}}_{\text{iLNb}}- {\mathrm{w}}_{\text{iLNa}}$ space with and without a prepulse. The black dots in Fig. 2b correspond to the values of ${\mathrm{w}}_{{\text{iLN}}{\text{b}}}$ and ${\mathrm{w}}_{\text{iLNa}}$ used in Fig. 2a. With our parameters, the n.r. region broadens due to the addition of a prepulse, indicating that the startle response is suppressed by a prepulse. Figure 2c shows the percentage of the surface area occupied by each behaviour in the ${\mathrm{w}}_{\text{iLNb}}- {\mathrm{w}}_{\text{iLNa}}$ space with and without a prepulse. Our simulations show that a prepulse leads to a decrease in the number of hunch responses and increases in the number of bend responses and n.r.

Matsumoto et al*.*^31^ did not distinguish between hunches and bends in the startle response. Thus, in this study, we performed behavioural experiments with wild-type *Canton-S* larvae and analysed the types of behaviours. As shown in Fig. 2d, a prepulse led to a decrease in hunching and increases in bending and n.r. (No prepulse (blue): hunch, 96.6 ± 1.3%, n = 30; bend, 3.4 ± 1.3%, n = 30; n.r., 0%, n = 30; with prepulse (red): hunch, 50.9 ± 6.2%, n = 38, ****p < 0.0001; bend, 29.7 ± 5.6%, n = 38, ***p < 0.001; n.r., 19.4 ± 4.3%, n = 38, **p < 0.01, Mann–Whitney U test). Thus, the experimental results show the changes in the percentage of each behaviour due to a prepulse, and these results were qualitatively consistent with the simulation results, showing that simulation using the neural circuit model could reproduce the experimental results.

### Reproduction of Jovanic et al.^32^ experimental results

Jovanic et al.^32^ demonstrated that their neural circuit model could predict changes in the startle response when the activities of particular neurons (iLNa and Hb) were silenced. We investigated whether the model with our adjusted parameters that induced PPI could reproduce the experimental results of silenced iLNa and Hb, as shown in Jovanic et al*.*^32^. In this case, the strength of the input stimulus to Ch neurons ${s}_{1}$ was set to 2.0, the same value as in Jovanic et al*.*^32^. A prepulse was not used in this section. Figure 2e,f show the percentage of the surface area of each behaviour in the ${\mathrm{w}}_{\text{iLNb}}-{\mathrm{w}}_{\mathrm{iLNa}}$ space when these neurons were silenced. Figure 2e shows the results when iLNa was silenced, and Fig. 2f shows the results when Hb was silenced. When iLNa was silenced, our simulation results were highly consistent with the experimental results of Jovanic et al*.*^32^ showing a considerable decrease in hunch responses and an increase in bend responses. When Hb was silenced, hunch responses increased and bend responses decreased in the behavioural experiment in Jovanic et al*.*^32^. Our simulation qualitatively showed the same trend (Fig. 2f), although our result values were slightly different than the experimental results in Jovanic et al*.*^32^. Accordingly, the adjustment of the parameters in our study did not change the properties of the neural circuit model developed by Jovanic et al*.*^32^; thus, the simulation in our research is valid.

### Relationship between PPI and cellular activity levels

**Figure 3:**
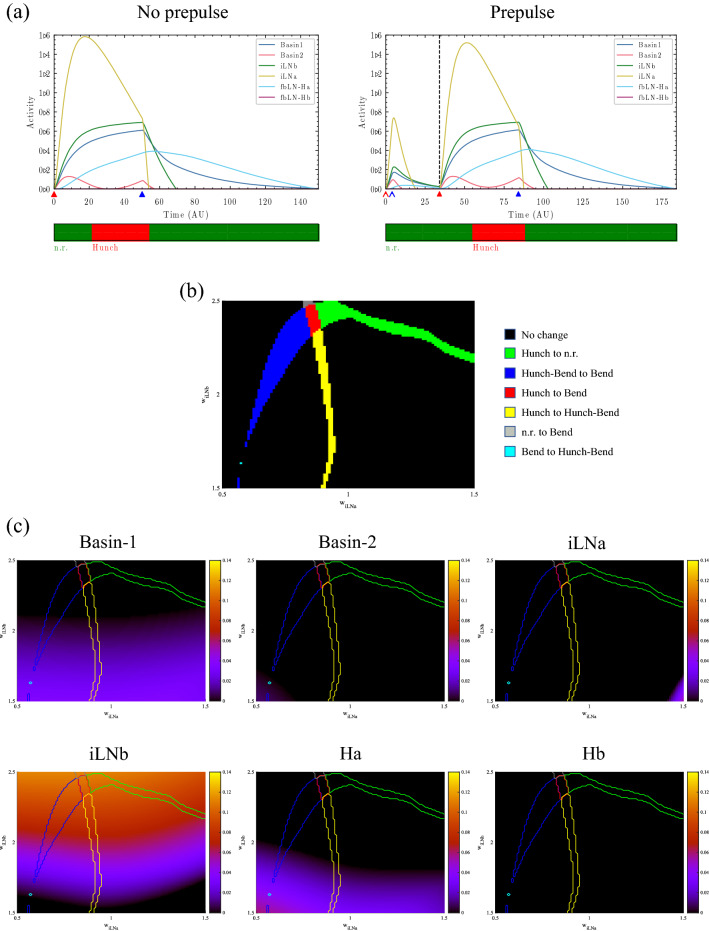
Relationship between PPI and cell activity. (**a**) Temporal changes in neuronal activity at the triangular dots shown in Fig. 2b (${\mathrm{w}}_{\text{iLNa}}=1.25,{\mathrm{w}}_{\text{iLNb}}=1.75$), where the startle response is not suppressed by a prepulse. Left: Without a prepulse; Right: With a prepulse. The vertical dashed black line indicates the time of the pulse input. The thin arrow head pointing to the horizontal axis indicates the prepulse timing, and the thick arrow head indicates the pulse timing. The red and blue colours indicate the onset and offset times, respectively. The colours in the band below each graph indicate the behaviour at the corresponding time. Green: n.r., red: hunch, blue: bend. (**b**) The behavioural landscape of behaviours in the ${\mathrm{w}}_{\text{iLNb}}- {\mathrm{w}}_{\text{iLNa}}$ space. Each colour indicates how the behaviour changed with and without a prepulse input. Black: no change. Red: hunch to n.r. Blue: hunch-bend sequence to bend. Red: hunch to bend. Yellow: hunch to hunch-bend sequence. Grey: n.r. to bend. Cyan: bend to hunch-bend sequence. (**c**) Persistence of each neuronal activity induced by a prepulse at the time of a pulse input as a ${\mathrm{w}}_{\text{iLNb}}- {\mathrm{w}}_{\text{iLNa}}$ spatial distribution. Each line indicates the boundary of behavioural changes due to PPI in (**b**). Orange colour indicates greater persistence of activity.

Under the conditions of PPI (Fig. 2a), iLNb activity induced by the prepulse remained during the pulse input, leading to a suppression of the hunch response. In this section, we investigated the relationship between PPI and the residual neural activities induced by a prepulse to clarify the factors underlying PPI. First, we examined the residual activities when PPI did not occur. The stimulus input time and pulse intensity were the same as in Sect. “Reproduction of PPI”. Figure 3a shows the typical behaviour of the circuit with our parameters in the case when the startle response was not suppressed by a prepulse (${\mathrm{w}}_{\text{iLNa}}=1.25,{\mathrm{w}}_{\text{iLNb}}=1.75$). These values correspond to the values of ${\mathrm{w}}_{\text{iLNb}}$ and ${\mathrm{w}}_{\text{iLNa}}$ represented by the triangular dots in Fig. 2b. Figure 3a (left) shows the results without a prepulse, and Fig. 3a (right) shows the results with a prepulse. Without a prepulse, iLNa activity directly after the stimulus input greatly exceeded iLNb activity. As a result, Basin-1 was activated more strongly than Basin-2, and a hunch response was evoked. With a prepulse, each neuronal activity induced by the prepulse reached baseline levels prior to the presentation of the startle stimulus. Thus, these activities showed the same dynamics as in the case without a prepulse, and the hunch response was evoked. The computational results when PPI occurred (Fig. 2a right) and when it did not occur (Fig. 3a right) suggested that the residual neural activities induced by a prepulse are a factor underlying PPI. Next, we investigated the entire ${\mathrm{w}}_{\text{iLNb}}- {\mathrm{w}}_{\text{iLNa}}$ space to broadly identify factors that induce PPI. Figure 3b shows the behavioural landscape of changes in the startle response due to a prepulse in the ${\mathrm{w}}_{\text{iLNb}}- {\mathrm{w}}_{\text{iLNa}}$ space. This plot shows the differences between the two diagrams in Fig. 2b. Figure 3c shows the persistence of neuronal activities induced by a prepulse at the time of the pulse input as a ${\mathrm{w}}_{{\text{i}}{\text{LNb}}}- {\mathrm{w}}_{\text{iLNa}}$ spatial distribution. In the region where the response changed from hunch to n.r. or bend due to PPI, as shown in Fig. 3b, the persistence of iLNb activity induced by a prepulse was significantly greater than that of other neurons (Fig. 3c). These results strongly indicate that the persistence of iLNb activity induced by a prepulse is a factor in the occurrence of PPI.

### Dependence of PPI on the lead interval

Matsumoto et al*.*^31^ reported that the effect of PPI depends on the time between a prepulse and a pulse, known as the lead interval. A lead interval between 0.3 and 0.5 s results in the strongest PPI effect. A shorter or longer lead interval led to a reduction in PPI^31^. We investigated the dependence of PPI on the lead interval using a computational model with our adjusted parameters. In this section, we set the stimulus input time and pulse intensity to the same value as in Sect. “Reproduction of PPI” while varying the lead interval.

**Figure 4:**
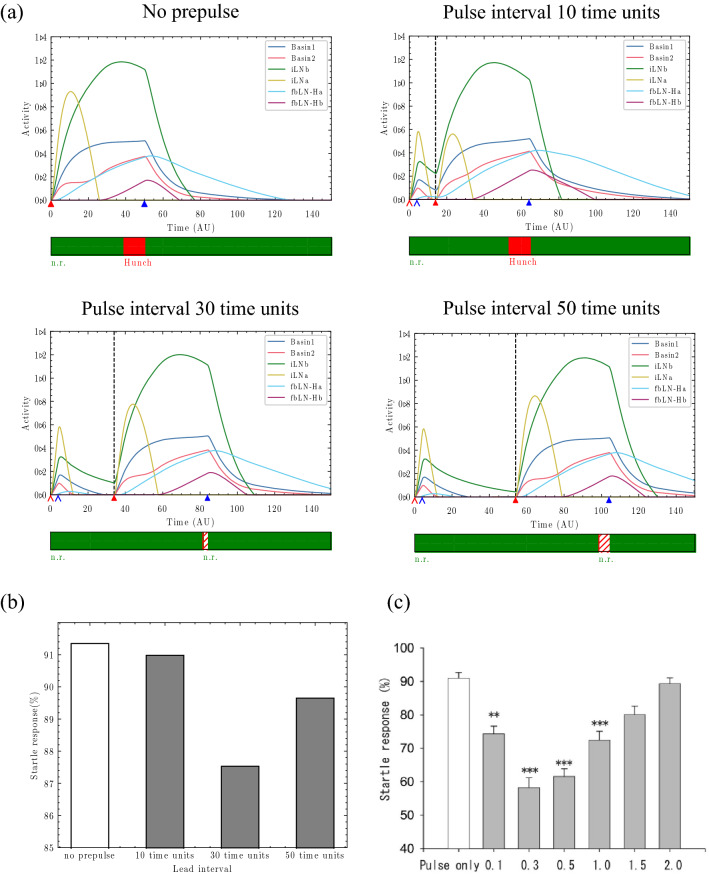
Dependence of PPI on the lead interval. (**a**) Temporal changes in neuron activity (${\mathrm{w}}_{\text{iLNa}}=1.10,{\mathrm{w}}_{{\text{iL}}{\text{Nb}}}=2.40$) as the lead interval changed. Upper left: Without a prepulse. Upper right: Lead interval of 10 time units. Lower left: Lead interval of 30 time units. Lower right: Lead interval of 50 time units. A vertical dashed black line indicates the time of the pulse input. The thin arrow head pointing to the horizontal axis indicates the prepulse timing, and the thick arrow head indicates the pulse timing. The red and blue colours indicate the onset and offset times, respectively. The colours in the band below each graph indicate the behaviour at the corresponding time. Green: n.r. Red: hunch. Behaviours indicated by the striped lines are classified as n.r. because their duration was shorter than the cut-off time (10 time units). (**b**) Changes in percentages of the startle response in the behavioural landscape due to changes in the lead interval. (**c**) Behavioural experimental results of changes in the startle response due to changes in the lead interval. Reprinted from Matsumoto et al*.*^31^ Fig. 1F.

Figure 4a shows the typical behaviours of the circuit when the lead interval was 10 time units, 30 time units, and 50 time units (${\mathrm{w}}_{\text{iLNa}}=1.10,{\mathrm{w}}_{\text{iLNb}}=2.40$). When the lead interval was 10 time units (Fig. 4a, upper right), the activity of neurons other than iLNb, such as Basin-1 and Ha, remained elevated at the time of the pulse input. Ha inhibited the activity of iLNb, which inhibited the activity Basin-1 and thus promoted the disinhibition of Basin-1. Moreover, Basin-1 was considerably more active than Basin-2 due to the residual activity of Basin-1. As a result, the hunch response was evoked without inducing PPI. When the lead interval was 30 time units or 50 time units (Fig. 4a, lower left and lower right), only the activity of iLNb remained elevated at the time of the pulse input, while the activity of Basin-1 was inhibited. In addition, the difference between the activities of Basin-1 and Basin-2 decreased, and the hunch response was suppressed, leading to n.r., as the time for satisfying the conditions of the hunch response (Eq. 1) was shorter than the cut-off time. Note that the comparison of 30 time units and 50 time units revealed that in the case of 50 time units, the inhibition of Basin-1 was weaker because the activity of iLNb was reduced at the time of the pulse input, as indicated by the vertical dashed black line. As a result, the duration of the response for satisfying the hunch condition (Eq. 1) was greater than that of 30 time units. This finding indicates that a lead interval of 50 time units is closer to the situation that evokes a startle response, representing a reduction in PPI. Therefore, PPI may be attenuated when activities other than iLNb remain elevated at the time of the pulse input or when the remaining iLNb activity is extremely low. Next, Fig. 4b shows the percentages of the surface area occupied by the startle response in the ${\mathrm{w}}_{\text{iLNb}}- {\mathrm{w}}_{\text{iLNa}}$ space as the lead interval changed. The effect of PPI was the greatest when the lead interval was 30 time units and weaker when the lead interval was 10 time units or 50 time units. This result is consistent with the experimental results of Matsumoto et al*.*^31^ (Fig. 4c) and indicates that PPI is dependent on the lead interval.

### Relationship between PPI and the Centaurin mutant

**Figure 5:**
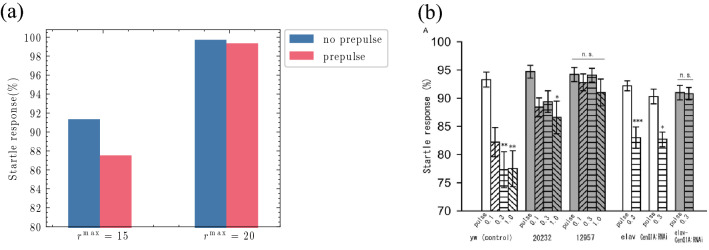
PPI in the CenG1A mutants. (**a**) Changes in the percentages of the startle response due to PPI when ${r}^{\text{max}}$, the maximum activity of all neurons, was changed. ${r}^{\text{max}}=15$ is considered to correspond to the activity level of wild-type individuals, and ${r}^{\text{max}}=20$ is considered to correspond to CenG1A mutants. (**b**) Results of behavioural experiments. The white bars show the results for the wild-type larvae, and the shaded bars show the results for the mutants. *12957* indicates the results of the CenG1A mutants. Reprinted from Matsumoto et al*.*^31^ Fig. 3A.

Matsumoto et al*.*^31^ investigated CenG1A, a protein that may be related to autism, and found that PPI was decreased in *Drosophila* larvae with loss of CenG1A function. Homma et al*.*^34^ found that neurotransmitter release was enhanced in larvae with suppressed CenG1A function. As mentioned above, we used simulations of the dependence of PPI on the lead interval to show that non-iLNb neuronal activity at the time of the pulse input was a factor in reducing PPI. These findings suggest that the following factor is involved in the deficiency of PPI in CenG1A mutants: Each type of cell activity in the neural circuit is enhanced by the suppression of CenG1A, which inhibits neurotransmitter release in neurons that express CenG1A. As a result, non-iLNb cell activities remain at the time of the pulse, leading to a reduction in PPI. To test this hypothesis, we conducted simulations using our mathematical model. Because CenG1A is ubiquitously expressed, its effects on neurotransmitters are considered to act not on specific neurons but on the entire neural circuit; thus, the effects can be reproduced by increasing ${r}^{\text{max}}$, the parameter for controlling the synaptic strength of the neurons in the mathematical model, which represents the maximum value of the neuronal activities. Accordingly, we set ${r}^{\text{max}}$ to be greater than our adjusted value (increasing ${r}^{\text{max}}=15$ to ${r}^{\text{max}}=20$). The stimulus input time and pulse intensity were set to the same values as in Sect. “Reproduction of PPI”. The simulation results are shown in Fig. 5. Figure 5a shows the change in the startle response due to PPI as ${r}^{\text{max}}$ changed. Compared with ${r}^{\text{max}}=15$, the suppression of the startle response was weakened when ${r}^{\text{max}}=20$. Thus, increasing the strength of the neural activities in the mathematical model reduced the effect of PPI. This change is qualitatively consistent with the behavioural results of the responses of the control animals (Fig. 5b, yw), which showed PPI, and CenG1A mutants (Fig. 5b, *12957*), which showed no PPI, as previously reported by Matsumoto et al*.*^31^. In addition, by examining each neuronal activity level, we found that the residual iLNb activity induced by the prepulse was reduced at the time of the pulse input, while the residual activities of the other neurons, including Basin-1 and Ha, were higher when ${r}^{\text{max}} =20$ than when ${r}^{\text{max}}=15$. These results support the validity of the hypothesis that the reduction in PPI in CenG1A mutants is due to residual neuronal activity because of increased transmitter release.

## Discussion

It is important to infer how neural networks function by using simulations with computational models and discussing the results in combination with experimental results. In this study, we reproduced our experimental PPI results and the behavioural experimental results of Jovanic et al*.*^32^ by adjusting the parameters of the neural circuit model reported in Jovanic et al*.*^32^. These results suggest the generalizability of the neural circuit model and the validity of the model after our parameter adjustment. By using the model to investigate temporal changes in cell activities associated with PPI, we found that iLNb inhibition, which is induced by a prepulse, is strongly involved in PPI. In addition, with the simulation results in this study, we validated speculations about the cause of PPI reduction in CenG1A mutants. We not only showed that a neural circuit model with adjusted parameters can explain the experimental results, including PPI, but also shed light on the cause of PPI reduction at the neuronal level through simulations.

The simulation results using the neural circuit model with our adjusted parameters reproduced the results of multiple behavioural experiments, suggesting the following mechanism for PPI. When iLNb activity induced by a prepulse input is higher than other cell activities, iLNb inhibits Basin-1 activity; at the same time, Basin-1 and Basin-2 are coactivated. This in turn induces PPI; the startle response is suppressed, and hunches are suppressed while bends are enhanced. On the other hand, when non-iLNb neuronal activities such as Basin-1 and Ha remain at the time of the pulse input, Ha suppresses iLNb activity and promotes the disinhibition of Basin-1; thus, PPI is suppressed because Basin-1 is more strongly activated than Basin-2 due to the residual activity of Basin-1. Based on the connectome and their experimental results, Jovanic et al*.*^32^ reported that iLNb disinhibits Basin-2 and induces coactivation of Basin-1 and Basin-2, evoking the bend response. Our simulation results showing that iLNb activity contributes to PPI do not contradict Jovanic et al*.*^32^ discussion on the circuit mechanism of the startle response. Furthermore, we investigated factors that modulate PPI, such as the lead interval. The results revealed that PPI is reduced when non-iLNb activities remain or when the remaining iLNb activity is extremely low at the time of the pulse input. Thus, we suggest a neural circuit mechanism in which residual iLNb activity induces PPI while non-iLNb neuronal activities inhibit PPI. PPI is believed to be a measure of sensorimotor gating, which is a system that controls sensory input by filtering stimuli with low relevance to prevent an overflow of information into the system. Our findings suggest that in *Drosophila* larvae, the balance between the activities of iLNb neurons and other neurons is a mechanism for filtering sensory stimuli.

Whether the effect of PPI is mediated via intrinsic circuitry that regulates the startle response or via extrinsic circuitry (e.g., the pedunculopontine nucleus efferents to the nucleus reticularis pontis caudalis in rats) is still being discussed^22^. While many rat studies support the latter hypothesis, the neural circuit mechanism of PPI suggested in this study is realized within the neural circuit that determines the startle response, which is an intrinsic circuit. Although the neural basis of PPI likely differs between *Drosophila* larvae and mammals, it is important for PPI research that intrinsic circuitry can reproduce not only PPI but also PPI properties, such as the dependence on the lead interval. In future work, the validity of the neural mechanism shown in this study should be verified with physiological experiments.

The reproductions of the experimental results using simulations yielded qualitatively consistent results in terms of the percentages of behaviour selection. However, there were some quantitative inconsistencies (Figs. 2c,e,f and 4b). Furthermore, we noticed that there is an increased startle response in the case of ${r}^{\text{max}}=20$ compared with that of ${r}^{\text{max}}=15$ in Fig. 5a. In the analysis of the mathematical model used in our research, the percentages of each behaviour in ${\mathrm{w}}_{\text{iLNb}}- {\mathrm{w}}_{\text{iLNa}}$ parameter space were determined. This analysis assumed that each neuronal group was uniformly distributed in the ${\mathrm{w}}_{\text{iLNb}}-{\mathrm{w}}_{\text{iLNa}}$ space. However, the ${\mathrm{w}}_{\text{iLNb}}-{\mathrm{w}}_{\text{iLNa}}$ distributions in the *Drosophila* larvae used in the experiments may not be uniform; thus, the experimental results may be biased in some regions in the ${\mathrm{w}}_{\text{iLNb}}- {\mathrm{w}}_{\text{iLNa}}$ space. It is possible that these biased distributions caused the quantitative inconsistencies. Rigorous quantitative reproduction may require narrower ${\mathrm{w}}_{\text{iLNb}}-{\mathrm{w}}_{\text{iLNa}}$ space simulations if this is the reason for the quantitative inconsistencies. It has also been reported that behaviour selection in a neural circuit is related to higher brain regions^36^. Because the mathematical model used in this study did not consider higher brain functions, this simplification may explain the quantitative differences with the behavioural experiment results. Further investigation using physiological and computational experiments will help to solve these quantitative discrepancies.

In this study, we adjusted the heuristically determined parameters in the mathematical model proposed by Jovanic et al*.*^32^ to reproduce our both PPI experimental results and Jovanic et al*.*^32^ mutant experiment results. Several parameters in Jovanic et al*.*^32^ and our studies, such as the time constant, were not set based on physiological experiments; instead, they were set manually by the researchers in a heuristic manner to reproduce the phenomenon. Thus, we cannot rule out the possibility that the parameters may have of different values and there may be other parameter regions that can reproduce these experimental results. However, the neural circuit mechanism of PPI shown in this paper is consistent with experimental results and discussions on PPI and neural circuits in *Drosophila* larvae. The results of the behavioural experiments, which were predicted and reproduced in Jovanic et al*.*^32^ were also reproduced by our model. This finding indicates that our adjusted parameters did not change the properties of the mathematical model, which validates our simulations. Furthermore, although our parameters were set to reproduce only the PPI experimental results and Jovanic et al*.*^32^ mutant experiments, the adjusted mathematical model reproduced the dependence of PPI on the lead interval, which is one of the important properties of PPI. This result differs from previous mathematical studies^27^, in which the model was developed to reproduce this property with some assumptions, demonstrating the validity of our model. Overall, although we cannot conclude that our parameters are the optimal values, the simulation results are consistent with the results of previous studies on PPI, and we believe that these values are promising candidates.

Next, we investigated factors that reduce PPI in CenG1A mutants. Our previous findings reported in Homma et al*.*^34^ suggested that the activity of each cell in the neural circuit was enhanced in CenG1A mutants because CenG1A functions as a negative regulator of neurotransmitter release. This may be the reason that cell activities other than iLNb remain elevated at the time of the pulse input in CenG1A mutants. Our results show with more certainty that CenG1A negatively regulates the neurotransmitter release mechanism in neurons expressing CenG1A. It has been reported that PPI is reduced in many psychiatric disorders, such as schizophrenia and autism. In addition, it has been reported that PPI is reduced in fragile X syndrome, an inherited intellectual disability disorder, with concomitantly, neural activity imbalance and excessive neural transmission also occurring^37^. We reported the loss of PPI in the fly model of fragile X syndrome^31^. Moreover, dopamine transmission has been reported to be elevated in patients with schizophrenia^38–41^. It is thus believed that maintaining neurotransmission in the proper range is critical for stabilizing neural circuits and modulating sensory and normal behaviours. Our study showed that even in a neural circuit involved in selecting a behavioural response to a simple sensory stimulus, excessive neural activity disrupts the behaviour selection. In many psychiatric disorders, changes in activity balance are believed to cause a variety of symptoms^42,43^. The findings of this study suggest that the activity level of iLNb is a key factor underlying PPI. Neural circuit simulations, as performed in this study, are expected to identify neurons that play a key role in maintaining neural activity balance at the cellular level.

## Data Availability

The datasets generated during and/or analysed during the current study are available from the corresponding author on reasonable request.
